# Hidden Challenges: A Cross-Sectional Study on Prevalence and Determinants of Sexual Dysfunction in Men and Women with Multiple Sclerosis

**DOI:** 10.3390/medicina62030522

**Published:** 2026-03-11

**Authors:** Desirèe Latella, Fabio Mauro Giambò, Gianluca La Rosa, Lilla Bonanno, Rocco Salvatore Calabrò

**Affiliations:** 1 Istituto di Ricovero e Cura a Carattere Scientifico (IRCCS), Centro Neurolesi Bonino-Pulejo, S.S. 113 Via Palermo, C.da Casazza, 98124 Messina, Italy; desiree.latella@irccsme.it (D.L.); roccos.calabro@irccsme.it (R.S.C.); 2 Department of Pneumology, AOU Policlinico Universitario in Messina, 98125 Messina, Italy; gianlucacarmelo.larosa@unime.it

**Keywords:** sexual dysfunction, multiple sclerosis, prevalence, quality of life

## Abstract

*Background and Objectives:* Sexual dysfunction (SD) is common in multiple sclerosis (MS) but remains under-recognized in routine care. This study aimed to quantify the burden of SD in men and women with relapsing–remitting MS (RRMS), describe sex-stratified patterns across primary/secondary/tertiary domains, and examine associations with fatigue and MS-related health-related quality of life (HRQoL). *Materials and Methods:* In this cross-sectional observational study, RRMS participants were voluntarily recruited online via a QR code linking to a Google Forms survey. Men completed the International Index of Erectile Function-5 (IIEF-5), and women the Female Sexual Function Index (FSFI). MS-specific SD domains were assessed using the Multiple Sclerosis Intimacy and Sexuality Questionnaire (MSISQ), alongside the Fatigue Severity Scale (FSS) and the Multiple Sclerosis Quality of Life questionnaire (MSQOL-54). Sex differences were tested using parametric/non-parametric methods as appropriate, with false discovery rate (FDR) and Bonferroni adjustments for multiple comparisons. *Results:* Thirty-seven participants were included (16 men; 21 women). Mean age did not differ by sex (35.9 ± 4.0 vs. 38.9 ± 10.4 years; *p* = 0.23). All participants reported at least some degree of difficulty across MSISQ domains. Among men, 87.5% screened positive for erectile dysfunction within this sample (mild 37.5%, mild-to-moderate 12.5%, moderate 12.5%, severe 25.0%). When dysfunction type was defined as the highest MSISQ domain score, secondary SD was most frequent in both sexes (75.0% men; 76.2% women; *p* = 0.49). Women showed higher secondary domain scores at the uncorrected level (*p* = 0.04), but this did not survive FDR correction. In HRQoL and symptom measures, women reported markedly higher fatigue (FSS 46.1 ± 12.4 vs. 25.5 ± 12.7; *p*_FDR < 0.001) and poorer physical health indices, including pain-related outcomes. *Conclusions:* SD has represented a substantial burden within this RRMS sample, with secondary domain predominance in both sexes, highlighting the clinical relevance of symptom-related and functional interference. These findings support the value of multidimensional sexual health assessment in clinical research settings and may be relevant for clinical assessment and future research in MS.

## 1. Introduction

Multiple sclerosis (MS) is a chronic inflammatory and neurodegenerative disease of the central nervous system that begins in early adulthood [1] during years in which sexuality, intimacy, and partnership often play a central role in quality of life. Sexual health remains a frequently underestimated domain and is often under-addressed in current MS care, despite its clear links to wellbeing and everyday functioning [2,3].

Sexual dysfunction (SD) in MS encompasses disturbances in desire, arousal, orgasm, and sexual satisfaction, and it can affect both women and men across disease phenotypes. Prevalence estimates are consistently high but vary widely across studies, largely due to differences in sampling and, crucially, in assessment tools and diagnostic thresholds. Recent meta-analyses suggest pooled prevalence estimates around 61% in women and 63% in men, while individual studies have reported broad ranges (17–95% and 31–92%, respectively), underscoring substantial between-study heterogeneity driven by sampling methods and assessment thresholds [4,5,6,7].

Within MS-specific conceptual models, SD is often described across three domains: primary dysfunction, reflecting direct neurogenic effects on sexual response (e.g., altered genital sensation, lubrication/erectile response, or orgasmic function) [8]; secondary dysfunction, reflecting indirect interference from MS-related symptoms and functional limitations (e.g., fatigue, pain, spasticity, sensory disturbances, bladder/bowel dysfunction, mobility limitations, and medication adverse effects) [9]; and tertiary dysfunction, reflecting psychological, emotional, and relational consequences of living with MS (e.g., changes in self-image, reduced confidence, avoidance, and relationship strain) [10]. This framework is clinically useful because it highlights that sexual difficulties in MS are frequently multidimensional and may require integrated assessments beyond a single symptom or mechanism [11].

However, many previous studies have examined these domains using either general sexual function scales or MS-specific instruments in isolation. As a result, it remains difficult to disentangle whether sexual complaints primarily reflect neurogenic impairment, symptom interference, or psychosocial adaptation. Combining sex-specific function measures (such as FSFI and IIEF-5) with an MS-specific multidomain instrument (MSISQ) and broader patient-reported outcomes may allow a more clinically informative characterization by distinguishing the severity of sexual symptoms from their contextual drivers. In particular, integrating fatigue and health-related quality-of-life measures could provide an opportunity to examine whether sexual difficulties align more closely with neurological disability per se or with global symptom burden.

From a neurobiological perspective, sexual function depends on distributed central nervous system networks integrating sensory, autonomic, endocrine, and cognitive–emotional processes. Lesions affecting spinal cord pathways, brainstem autonomic centers, and supraspinal regions involved in reward and motivation (including limbic and frontal circuits) may alter arousal, genital response, and sexual motivation. In MS, demyelinating and neurodegenerative processes can disrupt both ascending sensory input and descending autonomic regulation, contributing not only to impaired genital sensation and erectile or lubrication responses but also to altered interoceptive awareness and fatigue-related activity limitation. Consequently, sexual dysfunction in MS cannot be attributed solely to focal neurological damage as it often reflects the interaction between neural network disruption and broader symptom burden.

Beyond prevalence, research increasingly emphasizes determinants and clinical correlates of SD in MS, often implicating both neurological disability and modifiable factors such as fatigue and depression [12,13]. Observational works have underscored that SD is common in people with MS and meaningfully associated with fatigue and depression, while scoping evidence highlights consistent links with neuropsychiatric symptoms and relationship factors, as well as the comparatively limited and heterogeneous literature on cognition-related associations [14].

Despite increasing research interest, important uncertainties remain. This limits the interpretation of reported sex differences, as findings may depend on instrument selection rather than true clinical variability. Moreover, most available studies have focused either on prevalence estimation or on isolated predictors, whereas fewer have explored how sexual dysfunction relates to co-occurring symptom clusters such as fatigue and perceived physical health. Clarifying these relationships may have practical relevance for clinical assessment, as symptom-targeted management strategies could differ depending on whether sexual difficulties are primarily neurogenic or symptom-mediated. Reported prevalence and risk profiles are still difficult to compare across studies because different tools and non-uniform cut-offs can substantially change who is classified as having SD [15,16,17]. Moreover, the relative weight of neurogenic mechanisms versus symptom-mediated and psychosocial mechanisms varies across cohorts, and sex-specific profiles remain inconsistently described [16,18]. Finally, SD often remains under-reported and undertreated, with clinician-related barriers—including limited confidence and knowledge—contributing to the missed detection in routine care [19].

The present cross-sectional study aimed to quantify the burden of SD in men and women with relapsing–remitting MS (RRMS) and to describe sex-stratified patterns using a standardized assessment approach. Specifically, we estimate the prevalence and severity of SD and characterize symptom domains within the primary/secondary/tertiary framework, while exploring cross-sectional associations with fatigue and MS-related health-related quality of life, in an exploratory, hypothesis-generating framework. Given the exploratory nature of the study, a priori directional hypotheses were not formulated.

## 2. Materials and Methods

### 2.1. Study Design

This study employed a cross-sectional observational design to assess the prevalence and profile of SD in individuals with RRMS, and to explore associations with fatigue and health-related quality of life. Data were collected at a single timepoint using self-administered questionnaires delivered online. It was conducted between October 2025 and December 2025. The study was conducted in accordance with the Declaration of Helsinki and was approved by the local institutional ethics committee (approval number: CEL/U112/25). All participants provided electronic informed consent prior to accessing the questionnaires.

### 2.2. Participants and Recruitment

Participants were recruited online through dissemination of a study link generated via Google Forms. Access to the questionnaire battery was provided through a QR code redirecting to the online module. The questionnaire was accessible only after confirmation of informed consent and the completion of eligibility screening questions embedded at the beginning of the survey. The form was configured to allow a single submission per device and required the completion of mandatory fields to proceed to reduce incomplete responses. No financial or material incentives were provided for participation. Potential participants were screened within the form through eligibility questions; the respondents meeting the predefined criteria were included.

All eligibility criteria, including RRMS diagnosis, comorbidities, and exclusion conditions, were self-reported by participants and were not independently verified through medical records or clinician confirmation. This approach was chosen to facilitate participation and disclosure in a sensitive domain; however, it may reduce diagnostic verification compared with clinician-confirmed data.

A total of 41 individuals accessed the study after initial consent; 4 participants subsequently declined to complete the questionnaires, resulting in a final sample of 37 participants included in the analyses (completion rate: 90.2%). The final cohort comprised 16 men and 21 women, all diagnosed with RRMS. The survey required approximately 10–15 min to complete. Participants could review their responses before submission but could not modify their entries after final submission.

Inclusion criteria: (i) diagnosis of RRMS; (ii) completion of online informed consent; completion of the questionnaire battery.

Exclusion criteria: (i) age < 18 years; (ii) MS phenotype other than RRMS or unclear/unverifiable self-reported diagnosis; (iii) lack of electronic informed consent; (iv) incomplete questionnaires preventing calculation of total/subscale scores; (v) duplicate or inconsistent entries (when identified); (vi) pregnancy or recent postpartum period (e.g., within the previous 6 months), as self-reported; (vii) severe cognitive impairment or insufficient language comprehension likely to compromise reliable questionnaire completion, as self-reported; (viii) major psychiatric disorders not clinically stabilized (e.g., acute major depressive episode or severe anxiety disorder), as self-reported; (ix) major non-MS medical conditions directly affecting sexual function (urological/andrological/gynecological disorders), as self-reported; and (x) relevant endocrine/metabolic disorders not adequately controlled (e.g., untreated thyroid dysfunction or poorly controlled diabetes), as self-reported.

### 2.3. Procedures and Data Collection

After providing electronic informed consent, participants completed the questionnaires directly within the Google Forms environment. The survey was designed to be completed in one session and required no follow-up assessments. No repeated measures or longitudinal data collection were performed. Medication use (disease-modifying and symptomatic treatments) was not collected.

In accordance with the MS intimacy and sexuality questionnaire (MSISQ) conceptual model, MS-related SD was evaluated across three domains: primary dysfunction (direct neurogenic effects on sexual response); secondary dysfunction (indirect interference from MS-related symptoms and functional limitations that affect sexual activity); and tertiary dysfunction (psychological, emotional, and relational consequences of living with MS impacting sexuality).

Data were collected anonymously and no personally identifiable information was recorded. IP addresses were not stored. Responses were screened for duplicate entries and internal inconsistencies prior to analysis. Records with incomplete questionnaires preventing score calculation were excluded according to predefined criteria.

### 2.4. Outcome Measures

#### 2.4.1. Sexual Dysfunction—Sex-Specific Instruments

Men: Erectile/sexual function was assessed using the International Index of Erectile Function-5 (IIEF-5) [20], a validated screening tool for erectile dysfunction severity.

Women: Female sexual function was assessed using the Female Sexual Function Index (FSFI) [21], evaluating key domains of female sexual functioning.

#### 2.4.2. MS-Specific Sexual Dysfunction Profile

For both men and women, MS-related SD was additionally characterized using the MSISQ [22], which captured primary, secondary, and tertiary SD dimensions in MS.

#### 2.4.3. Health-Related Quality of Life

Health-related quality of life was assessed using the Multiple Sclerosis Quality of Life questionnaire (MSQOL) [23], providing a disease-relevant profile of perceived health status and functioning.

#### 2.4.4. Fatigue

Fatigue severity was measured using the Fatigue Severity Scale (FSS) [24], a commonly used instrument for fatigue burden in MS.

For all instruments, total and subscale scores were calculated according to the original authors’ scoring instructions. When permitted by the scoring manuals, missing single items within a scale were handled according to recommended procedures; otherwise, the questionnaire was considered incomplete and excluded from analysis.

### 2.5. Statistical Analysis

Sex differences were assessed both on continuous and categorical indicators of SD. For continuous variables, distributional assumptions were evaluated using the Shapiro–Wilk test in each sex separately.

When both distributions did not significantly deviate from normality, between-group comparisons were performed using *t*-tests; otherwise, Wilcoxon rank-sum tests were applied. For categorical variables (prevalent dysfunction type and high vs. low dysfunction), chi-square or Fisher’s exact tests were used depending on expected cell counts. To control for multiple testing, *p*-values were adjusted using the Benjamini–Hochberg false discovery rate (FDR) procedure within each family of related outcomes (sexual dysfunction domains and QoL measures). Erectile dysfunction severity was described in men using standard clinical categories. To characterize the predominant pattern of SD across sexes, dysfunction type was operationalized as the domain (primary, secondary, tertiary) with the highest subscale score for each participant. In parallel, absolute domain involvement was examined using an exploratory, data-driven classification of relative dysfunction severity. Because no universally accepted clinically validated cut-off scores are available for MSISQ domains, high dysfunction was defined as subscale scores greater than or equal to the median value of the entire sample for each domain. This approach was intended solely to allow within-sample comparisons and does not represent a diagnostic or clinically meaningful threshold. The resulting proportions of participants classified with relatively higher primary, secondary, and tertiary dysfunction were compared between sexes using categorical tests as described above. Two-sided *p*-values < 0.05 were considered statistically significant at the uncorrected level. All analyses were performed in R (version 4.2.2).

## 3. Results

### 3.1. Sample Characteristics and Sexual Dysfunction Prevalence

**Figure 1:**
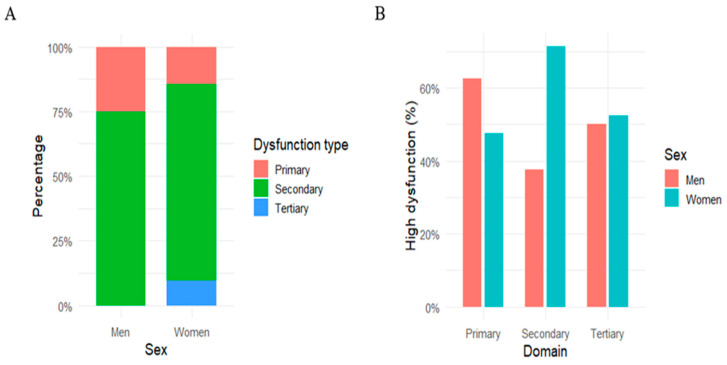
Sex-specific distribution of SD domains. (**A**) Proportion of men and women for whom primary, secondary, or tertiary dysfunction constitutes the dominant domain (prevalent dysfunction type). (**B**) Prevalence of high dysfunction in each domain by sex.

A total of 37 patients were included in the analysis (16 men and 21 women). Men had a mean age of 35.9 ± 4.0 years, whereas women were slightly older (38.9 ± 10.4 years) with no significant between-sex difference (*p* = 0.23). Mean disease duration was 11.9 ± 7.4 years in men and 9.2 ± 11.7 years in women (*p* = 0.04; *p*_FDR = 0.08). All participants showed non-zero scores in the primary, secondary, and tertiary SD domains, indicating that some degree of SD was present in the entire sample. Among men, 87.5% presented erectile dysfunction of any severity, with 37.5% classified as mild, 12.5% as mild-to-moderate, 12.5% as moderate, and 25.0% as severe, while only 12.5% reported no erectile dysfunction. When the SD type was defined as the domain with the highest subscale score (primary, secondary, or tertiary), secondary dysfunction was the most frequent prevalent subtype in both sexes (75.0% of men and 76.2% of women), followed by primary dysfunction (25.0% vs. 14.3%, respectively) and tertiary dysfunction (0% vs. 9.5%) (Figure 1A). Notably, the 0% prevalence of tertiary dysfunction among men reflected the absence of tertiary dysfunction as the dominant domain, not the absence of tertiary dysfunction per se, which was present in all participants. The distribution of prevalent dysfunction types did not differ significantly between men and women (Fisher’s exact *p* = 0.49). When comparing continuous domain scores between sexes, women showed higher secondary dysfunction scores than men (median 18.0 vs. 11.5), with a significant difference at the uncorrected level (*p* = 0.04), which did not survive FDR correction (*p*_FDR = 0.07). Primary and tertiary dysfunction scores did not differ between sexes (*p* = 0.55, *p*_FDR = 0.58 and *p* = 0.74, *p*_FDR = 0.77, respectively). Using a data-driven definition of high dysfunction based on the median of the whole sample, high primary, secondary, and tertiary dysfunction were common in both sexes (primary: 62.5% of men vs. 47.6% of women; secondary: 37.5% vs. 71.4%; tertiary: 50.0% vs. 52.4%) (Figure 1B). However, the prevalence of high dysfunction did not differ significantly between men and women for any domain (high primary: χ^2^ *p* = 0.57, *p*_FDR = 0.86; high secondary: χ^2^ *p* = 0.08, *p*_FDR = 0.25; high tertiary: χ^2^ *p* = 0.99, *p*_FDR = 0.99).

### 3.2. Quality of Life and Fatigue

Beyond sexual domains, several MS-related quality-of-life measures differed between sexes (Table 1 and Supplementary Table S1). At the uncorrected level, men showed significantly higher scores than women on physical health (*p* = 0.02), physical function (*p* = 0.02), role limitation—physical (*p* = 0.02), pain (*p* < 0.001), change in health (*p* = 0.03), social function (*p* = 0.02), health distress (including Health_Distress_2 and Health_Distress_3) (*p* = 0.02), energy (*p* = 0.004), health perceptions (*p* = 0.008), the energy/fatigue index (*p* = 0.004), and the physical health composite score (*p* = 0.009). Women, in contrast, reported markedly higher fatigue on the Fatigue Severity Scale (Tot_FSS; *p* < 0.001). After controlling for multiple comparisons using FDR, sex differences remained significant for pain (*p*_FDR < 0.001), energy (*p*_FDR = 0.03), energy/fatigue (*p*_FDR = 0.03), health perceptions (*p*_FDR = 0.04), the physical health composite score (*p*_FDR = 0.04), and Tot_FSS (*p*_FDR < 0.001), whereas all other quality-of-life domains showed only uncorrected between-sex differences that did not survive FDR correction.

## 4. Discussion

In this cross-sectional study, we investigated the multidimensional profile of SD in men and women with RRMS and explored its associations with symptom burden and health-related quality of life. Given the study design, findings should be interpreted as correlational and hypothesis-generating rather than indicative of directional or causal relationships. Three main findings emerged: First, sexual symptoms were highly prevalent in the cohort, with all participants reporting some degree of impairment in the primary, secondary, and tertiary MSISQ domains, and with most men screening positive for erectile dysfunction (ED). Second, when we identified the predominant MSISQ domain for each participant (i.e., the domain with the highest subscale score), secondary dysfunction was the most frequent profile in both sexes (≈75%). Third, sex differences were more pronounced in global symptom-related outcomes—particularly fatigue and physical health indices—than in sexual domain severity per se.

Given the exploratory nature of the analyses, these interpretations should be viewed as descriptive patterns within the present sample rather than mechanistic explanations.

### 4.1. Prevalence and Predominant Pattern of Sexual Dysfunction

The high proportion of men screening positive for ED was consistent with evidence indicating that ED is among the most frequent male sexual problems in MS, although prevalence estimates vary substantially across studies depending on instruments, thresholds, and recruitment setting [4]. In our sample, the ED estimate should be interpreted cautiously given the small male subsample size and the online recruitment strategy, which may have increased participation among individuals with relevant symptoms. At the same time, online self-reporting procedures may have facilitated disclosures in a domain that is often under-reported in routine clinical encounters, potentially improving symptom ascertainment.

The predominance of secondary dysfunction provides a clinically informative signal. This categorization reflected a relative distribution within the present sample and should not be interpreted as a clinically validated threshold. Rather than implying that neurogenic or psychosocial mechanisms were absent, it suggests that, within this RRMS cohort, sexual difficulties were most often framed by symptom-related and functional interference in everyday life [25]. This interpretation is supported by the broader outcome profile observed here, including markedly higher fatigue in women and persistent sex differences in key physical health indices (notably pain). While causality cannot be inferred from a cross-sectional design, these convergent findings could suggest that symptom burden and physical wellbeing are closely associated with sexual difficulties and may help contextualize the predominance of secondary domain involvement within this cohort. However, part of this pattern may also reflect medication-related effects, which could not be disentangled in the present design.

### 4.2. Sex Differences and Symptom Burden

A key nuance is that the “dominant” MSISQ domain does not imply that other domains are irrelevant. For example, tertiary symptoms were present to some degree in all participants even though tertiary dysfunction was never the highest-scoring domain among men. In addition, when we applied a sample-derived classification of “high dysfunction,” we did not observe significant sex differences across domains; however, such internal thresholds are primarily descriptive and future studies should confirm these patterns using clinically established cut-offs to improve comparability across cohorts.

Sex-related contrasts in MSISQ domain scores were modest and did not remain robust after correction for multiple testing, whereas sex differences in symptom-related outcomes were more consistent. This pattern may reflect limited statistical power rather than the absence of meaningful differences. Women reported markedly higher fatigue and poorer physical health indices, including worse pain-related scores (with higher MSQOL-54 scores indicating better HRQoL). It should be noted that in MSQOL-54, lower scores indicate poorer perceived health status. Therefore, very low values observed in some physical health domains likely reflect substantial perceived impairment rather than absence of symptoms. In a small sample, extreme values may also be influenced by inter-individual variability and potential floor effects and should be interpreted cautiously.

### 4.3. Clinical Implications

Clinically, this pattern is consistent with the possibility that symptom burden and secondary domain sexual difficulties frequently co-occur in RRMS, while pain and discomfort may limit engagement and satisfaction. Similar associations between fatigue/quality of life and SD have been reported in MS cohorts [26]. Accordingly, sexual health assessment in MS should extend beyond sex-specific function measures alone and be integrated with the routine management of fatigue, pain, and other modifiable contributors, alongside medication reviews and rehabilitative strategies when symptom-related interference predominates [27].

Our findings reinforce the need for systematic sexual health screening in MS care, as sexual problems are frequently under-addressed and may remain undisclosed unless clinicians proactively ask. A pragmatic approach could combine a brief sexual function screener, an MS-specific domain tool to contextualize the predominant drivers of dysfunction, and targeted follow-up focused on modifiable contributors (fatigue and pain management, bladder/bowel symptom strategies where relevant, medication review, and psychosocial support). One possible interpretation is that secondary domain dysfunction may emerge through a cascade in which fatigue and pain reduce physical and emotional engagement, leading to diminished sexual desire and potential relationship strain. In such cases, an interdisciplinary approach may be helpful. The neurologist may review disease activity and medications that exacerbate fatigue or sexual side effects. The physiotherapist may address deconditioning, energy conservation strategies, and pain management to improve functional capacity. The psychologist or sex therapist may work on communication, expectation adjustment, and coping strategies within the couple. These coordinated interventions do not target sexual function directly but rather the modifiable contributors that interfere with intimacy. These findings suggest that interdisciplinary care may be considered in patients with prominent secondary domain involvement. Importantly, embedding sexual health assessment within routine MS evaluations may not only improve symptom management but also enhance patient–clinician communication and overall health-related quality of life, supporting a more holistic and gender-sensitive model of MS care [7].

### 4.4. Strengths and Limitations

Key strengths of this study include the combined use of sex-specific instruments and an MS-specific multidomain framework, which allowed clinically meaningful profiling of SD beyond a single prevalence estimate. This integrative approach facilitated the examination of distinct contributors to sexual difficulties and enhanced the interpretability of findings in a clinical context.

Several limitations should also be acknowledged. The relatively small sample size, restriction to a relapsing–remitting MS cohort, online recruitment strategy, and cross-sectional self-report design limit generalizability. The voluntary online recruitment strategy may have also introduced selection bias, potentially over-representing individuals who were experiencing or concerned about sexual difficulties. Consequently, the reported prevalence estimates should not be interpreted as population-level rates but rather as descriptive findings within a self-selected sample. The cross-sectional design precludes any inference regarding temporal or causal relationships between sexual dysfunction, fatigue, and quality-of-life outcomes; therefore, observed associations should be interpreted as exploratory and hypothesis-generating. In addition, the reliance on self-reported diagnosis, comorbidities, and exclusion criteria may introduce misclassification of bias and residual confounding, as clinical information was not independently verified. Although online self-reporting may enhance disclosure in sensitive domains such as sexual health, it may also reduce diagnostic precision. Another important limitation is the lack of systematic control for medication use. Both disease-modifying therapies and symptomatic treatments (e.g., antidepressants, antispastics, medications for fatigue, and antihypertensives) may directly affect sexual function. Consequently, pharmacological effects may have contributed to the observed sexual difficulties and could represent an unmeasured confounding factor, particularly when interpreting the predominance of secondary domain dysfunction. In addition, several observed between-sex differences did not remain significant after correction for multiple comparisons, suggesting that larger, adequately powered samples are required to determine whether modest sex-related contrasts are robust and reproducible. Given the limited sample size, the possibility of type II error cannot be excluded, and non-significant findings should therefore be interpreted cautiously as potentially inconclusive rather than definitively null.

Overall, these findings should be interpreted in light of the exploratory design, the limited sample size, and the reliance on self-reported clinical information. Accordingly, the results are best viewed as preliminary and hypothesis-generating rather than confirmatory.

### 4.5. Future Directions

Building on these findings, future research should prioritize larger, multicenter cohorts with standardized clinical characterization (e.g., disability metrics and disease activity) and longitudinal designs to clarify temporal relationships between symptom trajectories—particularly fatigue and pain—and specific domains of SD. Stratified analyses according to disease-modifying therapies and concomitant medications will be essential to disentangle treatment-related effects. In addition, broader assessment of psychosocial variables, including mood, relationship functioning, and partner-reported outcomes, is needed to better capture the interpersonal and quality-of-life impact of SD in MS.

## 5. Conclusions

In conclusion, SD in RRMS appears to be a multidimensional outcome emerging from the interplay between neurological disease processes, symptom-related interference, and psychosocial factors. The importance of secondary domain involvement highlights the association between the relationship between sexual difficulties and symptom burden, particularly fatigue and pain, within this cohort. Given the heterogeneity in assessment approaches and thresholds across the literature, future studies should prioritize larger samples, standardized clinical characterization, and clinically validated cut-offs, within longitudinal designs capable of clarifying links between symptoms and sexual outcomes.

## Figures and Tables

**Table 1:** Comparison of SD domains and health-related quality-of-life measures between men and women.

	Men (Mean ± SD)	Women (Mean ± SD)	*p*-Value	*p*-Adjusted
Primary sexual dysfunction	12.25 ± 5.26	11.05 ± 6.10	0.55 ^¥^	0.58
Secondary sexual dysfunction	14.13 ± 6.23	18.67 ± 7.22	0.04 *^¥^	0.07
Tertiary sexual dysfunction	9.50 ± 3.39	11.57 ± 6.83	0.74 ^¥^	0.77
Physical health	75.63 ± 27.38	50.00 ± 32.98	0.02 *^¥^	0.06
Role limitation physical	65.63 ± 46.44	28.57 ± 42.05	0.02 *^¥^	0.06
Role limitation emotional	54.17 ± 48.50	31.75 ± 42.79	0.16 ^¥^	0.20
Bodily pain	77.71 ± 22.79	6.19 ± 6.57	<0.001 *^¥^	<0.001 *
Emotional wellbeing	69.50 ± 15.86	54.29 ± 24.71	0.05 ^¥^	0.09
Energy	52.00 ± 18.82	33.14 ± 17.58	0.004 *^×^	0.03 *
Cognitive function	68.13 ± 20.97	52.86 ± 28.09	0.07 ^×^	0.09
Health distress	77.50 ± 19.66	54.29 ± 25.95	0.03 *^¥^	0.06
Sexual function	61.46 ± 31.90	69.44 ± 32.85	0.33 ^¥^	0.37
Change in health	56.25 ± 25.00	35.71 ± 24.46	0.03 *^¥^	0.07
Satisfaction with sexual function	59.38 ± 28.69	58.33 ± 35.65	0.99 ^¥^	0.99
Overall quality of life	31.24 ± 12.28	26.25 ± 15.22	0.19 ^¥^	0.23
Physical function	12.86 ± 4.65	8.50 ± 5.61	0.02 *^¥^	0.06
Health perceptions	9.56 ± 4.56	5.91 ± 2.35	0.01 *^×^	0.05
Energy fatigue	6.24 ± 2.26	3.98 ± 2.11	0.004 *^×^	0.03
Role limitation physical	7.88 ± 5.57	3.43 ± 5.05	0.02 *^¥^	0.06 ^¥^

^×^ *t*-test; ^¥^ Wilcoxon rank-sum test; * *p* < 0.05; *p*-adjusted: Benjamini–Hochberg FDR-adjusted *p*-values.

## Data Availability

The datasets generated and analyzed during the current study are available from the corresponding author upon reasonable request.

## Supplementary Materials

The following supporting information can be downloaded at: https://www.mdpi.com/article/10.3390/medicina62030522/s1, Table S1. Additional QoL and derived measures Comparison comparing men and women.

## Abbreviations

The following abbreviations are used in this manuscript:

MS	Multiple Sclerosis
SD	Sexual Dysfunction
RRMS	Relapsing–Remitting Multiple Sclerosis

## References

[1] Jakimovski D., Bittner S., Zivadinov R., A Morrow S., Benedict R.H., Zipp F., Weinstock-Guttman B. (2024). Multiple sclerosis. Lancet.

[2] Li V., Haslam C., Pakzad M., Brownlee W.J., Panicker J.N. (2020). A practical approach to assessing and managing sexual dysfunction in multiple sclerosis. Pract. Neurol..

[3] Marck C.H., Jelinek P.L., Weiland T.J., Hocking J.S., De Livera A.M., Taylor K.L., Neate S.L., Pereira N.G., Jelinek G.A. (2016). Sexual function in multiple sclerosis and associations with demographic, disease and lifestyle characteristics: An international cross-sectional study. BMC Neurol..

[4] Dastoorpoor M., Zamanian M., Moradzadeh R., Nabavi S.M., Kousari R. (2021). Prevalence of sexual dysfunction in men with multiple sclerosis: A systematic review and meta-analysis. Syst. Rev..

[5] Azimi A., Hanaei S., Sahraian M.A., Mohammadifar M., Ramagopalan S.V., Ghajarzadeh M. (2019). Prevalence of Sexual Dysfunction in Women with Multiple Sclerosis: A Systematic Review and Meta-Analysis. Maedica.

[6] Yazdani A., Ebrahimi N., Mirmosayyeb O., Ghajarzadeh M. (2023). Prevalence and risk of developing sexual dysfunction in women with multiple sclerosis (MS): A systematic review and meta-analysis. BMC Women’s Health.

[7] Di Pauli F., Zinganell A., Böttcher B., Walde J., Auer M., Barket R., Berek K., Egger A., Griesmacher A., Sukalo N. (2023). Sexual dysfunction in female and male people with multiple sclerosis: Disability, depression and hormonal status matter. Eur. J. Neurol..

[8] Calabrò R.S. (2018). Sexual dysfunction in Neurological disorders: Do we see just the tip of the iceberg?. Acta Biomed..

[9] Gustavsen S., Olsson A., Søndergaard H.B., Andresen S.R., Sørensen P.S., Sellebjerg F., Oturai A. (2021). The association of selected multiple sclerosis symptoms with disability and quality of life: A large Danish self-report survey. BMC Neurol..

[10] Bass A.D., Van Wijmeersch B., Mayer L., Mäurer M., Boster A., Mandel M., Mitchell C., Sharrock K., Singer B. (2020). Effect of Multiple Sclerosis on Daily Activities, Emotional Well-being, and Relationships: The Global vsMS Survey. Int. J. MS Care.

[11] Foley F.W., Zemon V., Campagnolo D., Marrie R.A., Cutter G., Tyry T., Beier M., Farrell E., Vollmer T., Schairer L. (2013). The Multiple Sclerosis Intimacy and Sexuality Questionnaire—Re-validation and development of a 15-item version with a large US sample. Mult. Scler..

[12] Pöttgen J., Rose A., Van De Vis W., Engelbrecht J., Pirard M., Lau S., Heesen C., Köpke S. (2018). Sexual dysfunctions in MS in relation to neuropsychiatric aspects and its psychological treatment: A scoping review. PLoS ONE.

[13] Maier S., Bajkó Z., Roșescu R., Bărcuțean L., Sărmășan E., Voidăzan S., Bălașa R. (2023). Sociodemographic and Clinical Determinants of Fatigue in Multiple Sclerosis. Life.

[14] Calabrò R.S., Russo M. (2015). Sexual Dysfunction and Depression in Individuals with Multiple Sclerosis: Is there a Link?. Innov. Clin. Neurosci..

[15] Hayes R.D., Bennett C.M., Dennerstein L., Taffe J.R., Fairley C.K. (2008). Are aspects of study design associated with the reported prevalence of female sexual difficulties?. Fertil. Steril..

[16] Petracca M., Carotenuto A., Scandurra C., Moccia M., Rosa L., Arena S., Ianniello A., Nozzolillo A., Turrini M., Streito L. (2023). Sexual dysfunction in multiple sclerosis: The impact of different MSISQ-19 cut-offs on prevalence and associated risk factors. Mult. Scler. Relat. Disord..

[17] DeRogatis L.R., Burnett A.L. (2008). The Epidemiology of Sexual Dysfunctions. J. Sex. Med..

[18] Culig L., Chu X., Bohr V.A. (2022). Neurogenesis in aging and age-related neurodegenerative diseases. Ageing Res. Rev..

[19] Al-Shaiji T.F. (2022). Breaking the Ice of Erectile Dysfunction Taboo: A Focus on Clinician-Patient Communication. J. Patient Exp..

[20] Rosen R., Cappelleri J., Smith M., Lipsky J., Peña B. (1999). Development and evaluation of an abridged, 5-item version of the International Index of Erectile Function (IIEF-5) as a diagnostic tool for erectile dysfunction. Int. J. Impot. Res..

[21] Wiegel M., Meston C., Rosen R. (2005). The Female Sexual Function Index (FSFI): Cross-Validation and Development of Clinical Cutoff Scores. J. Sex. Marital. Ther..

[22] Çuvadar A., Güneş A., Baş Y.Ç., Kehaya S. (2025). Determining the Effects of Emotional Freedom Techniques on Sexual Dysfunction and Self-Care Management in Women Diagnosed with Multiple Sclerosis. Brain Behav..

[23] Vickrey B.G., Hays R.D., Harooni R., Myers L.W., Ellison G.W. (1995). A health-related quality of life measure for multiple sclerosis. Qual. Life Res..

[24] Ruotolo I., Sellitto G., Ianniello A., Petsas N., Castelli L., Galeoto G., Berardi A., Barletta V., Conte A., Pozzilli C. (2022). Italian translation and validation of fatigue symptoms and impacts questionnaire in relapsing multiple sclerosis (FSIQ-RMS). Neurol. Sci..

[25] Totuk O., Türkkol M., Yadi F., Güdek H.C., Erci G.Ç., Doğan İ.G., Tezer D.Ç., Sahin S., Demir S. (2025). Sexual dysfunction in multiple sclerosis: Prevalence, risk factors, and impact on quality of life in a large cohort study. BMC Neurol..

[26] Hirt J., Dembowska K., Woelfle T., Axfors C., Granziera C., Kuhle J., Kappos L., Hemkens L.G., Janiaud P. (2024). Clinical trial evidence of quality-of-life effects of disease-modifying therapies for multiple sclerosis: A systematic analysis. J. Neurol..

[27] Nazari F., Shaygannejad V., Mohammadi Sichani M., Mansourian M., Hajhashemi V. (2020). Sexual dysfunction in women with multiple sclerosis: Prevalence and impact on quality of life. BMC Urol..

