# Supplementary material for: Hidden Challenges: A Cross-Sectional Study on Prevalence and Determinants of Sexual Dysfunction in Men and Women with Multiple Sclerosis
Source: Medicina (Kaunas). 2026 Mar 11;62(3):522. doi: 10.3390/medicina62030522 (PMC13027831; doi:10.3390/medicina62030522)
Supplement: Supplementary file 1 [file medicina-62-00522-s001.zip › medicina-4141751-supplementary.pdf]

medicina

MDPI

Table S1. Additional QoL and derived measures Comparison comparing men and women.

	Men (Mean ± SD)	Women (Mean ± SD)	*p*-Value	*p*-Adjusted
Pain_2	8.55 ± 2.51	6.43 ± 3.12	0.05 ¥	0.08
Sexual_Function_2	4.92 ± 2.55	5.56 ± 2.63	0.33 ¥	0.37
Social_Function	9.13 ± 2.33	7.48 ± 1.97	0.02 *¥	0.06
Health_Distress_2	8.53 ± 2.16	5.97 ± 2.85	0.03 *¥	0.06
Physical_Health_Composite	67.65 ± 20.54	47.25 ± 15.74	0.01 *¥	0.04 *
Health_Distress_3	10.85 ± 2.75	7.60 ± 3.63	0.03 *¥	0.06
Overall_QoL_3	5.62 ± 2.21	4.72 ± 2.74	0.19 ¥	0.23
Emotional_well_belling_3	20.16 ± 4.60	15.74 ± 7.17	0.05 ¥	0.09
Role_Limitation_emotional_3	13.00 ± 11.64	7.62 ± 10.27	0.16 ¥	0.20
Cognitive_Function_3	10.22 ± 3.14	7.93 ± 4.21	0.07 ×	0.09
Mental_Health_Composite	59.85 ± 21.12	43.62 ± 22.79	0.08 ¥	0.11
Fatigue Severity Scale	25.50 ± 12.66	46.10 ± 12.35	<0.001 *×	<0.001 *

× *t*-test; ¥ Wilcoxon rank-sum test; * $p < 0.05$; *p*-adjusted: Benjamini–Hochberg FDR-adjusted *p*-values.
